# Conversion of Low-Flow Priapism to High-Flow State Using T-Shunt with Tunneling

**DOI:** 10.1155/2017/7394185

**Published:** 2017-02-26

**Authors:** Neil A. Mistry, Nicholas N. Tadros, Jason C. Hedges

**Affiliations:** ^1^School of Medicine, Oregon Health & Science University, 3303 SW Bond Ave. CH10U, Portland, OR 97239, USA; ^2^Department of Urology, Oregon Health & Science University, Portland, OR, USA

## Abstract

*Introduction*. The three types of priapism are stuttering, arterial (high-flow, nonischemic), and venoocclusive (low-flow, ischemic). These are usually distinct entities and rarely occur in the same patient. T-shunts and other distal shunts are frequently combined with tunneling, but a seldom recognized potential complication is conversion to a high-flow state.* Case Presentation*. We describe 2 cases of men who presented with low-flow priapism episodes that were treated using T-shunts with tunneling that resulted with both men having recurrent erections shortly after surgery that were found to be consistent with high-flow states. Case 1 was a 33-year-old male with sickle cell anemia and case 2 was a 24-year-old male with idiopathic thrombocytopenic purpura. In both cases the men were observed over several weeks and both men returned to normal erectile function.* Conclusions*. Historically, proximal shunts were performed only in cases when distal shunts failed and carry a higher risk of serious complications. T-shunts and other distal shunts combined with tunneling are being used more frequently in place of proximal shunts. These cases illustrate how postoperative erections after T-shunts with tunneling can signify a conversion from low-flow to high-flow states and could potentially be misdiagnosed as an operative failure.

## 1. Introduction

Priapism is defined as a complete or partial penile erection not associated with stimulation, which persists for 4 hours or more beyond any sexual stimulation [1]. Priapism cases are emergencies that manifest from a loss of circulatory regulation (typically) in the corpora cavernosa. Failure to urgently evaluate and treat priapism can result in ischemic necrosis and complete loss of function of the penile tissue [2].

The three types of priapism are low-flow, stuttering, and high-flow priapism. Low-flow priapism—also called venoocclusive or ischemic priapism—is caused by blocked venous outflow that prevents oxygenated, arterial blood from perfusing the corpora cavernosa. This form of compartment syndrome presents with a persistent, rigid, and painful erection. The lack of arterial inflow creates a hypoxic environment that damages the smooth muscle tissue of the corpora cavernosa and can eventually lead to irreversible necrosis and fibrosis. Stuttering or intermittent priapism is generally limited to men with hematological malignancies such as sickle cell disease in which the patient suffers from multiple episodes of painful erections that vary in frequency and duration and often lead to ischemic priapism. Patients with previous ischemic priapism are also at risk for developing stuttering priapism. High-flow or nonischemic priapism is characterized by normal outflow with unregulated inflow of blood into the lacunar spaces that bypasses the regulatory helicine arteriolar beds [1]. The most common etiology is arterial-sinusoidal fistulas leading to unregulated inflow. While the penis presents as tumescent, it is often neither fully erect nor painful [3].

The American Urological Association describes a stepwise approach to the management of priapism beginning with intracorporal aspiration and injections of sympathomimetic agents such as phenylephrine. As a second-line therapy, distal cavernoglanular (corporoglandular) shunts are indicated as the first choice among shunting procedures due to their relative ease and reduced complications. Proximal shunting procedures such as the Quackles, Grayhack, or Barry shunts can be considered when distal shunting methods fail and circulation is not reestablished. These procedures are more technically challenging and come with higher risks for erectile dysfunction. They also require an operating room setting and are more difficult for the occasional practitioner to perform. There are reports of more serious adverse events after proximal shunts including urethral fistulae and purulent cavernositis after a Quackles shunt [4] and pulmonary embolism following the Grayhack procedure [5].

Among the various techniques for creating a distal shunt, the T-shunt procedure, a modification of the Al-Ghorab shunt, termed the Burnett “snake” maneuver has been described that involves tunneling the corporal bodies through the distal tunical defect with a Hegar dilator to facilitate better corporal drainage [6, 7]. A growing body of literature suggests that modified distal shunt techniques can offer excellent erectile function and minimize complications. Long-term results from this technique have shown that this technique can resolve ischemic priapism refractory to first- and second-line treatments and can also prevent further episodes of priapism.

The AUA guideline panel had difficulty determining the actual risks and benefits of proximal versus distal shunts from the literature due to the fact that patients frequently received multiple treatments and it was difficult to determine which treatment actually caused the adverse events. A recent study by Zacharakis et al. using 45 patients undergoing distal shunting with tunneling between 2009 and 2012 demonstrated 100% detumescence on the operating table. Recurrence varied depending on the duration of the priapism with permanent resolution in all patients with priapism lasting less than 24 hours and 55% of patients with original priapism lasting between 24–48 hours [8]. Tabibi et al. showed equal rates of erectile dysfunction between proximal and distal shunts in their population of 16 patients from 1995 to 2005 [9].

These modified distal shunt procedures have proven to be highly effective monotherapies for priapism, even if the erection is of extended duration [10, 11]. With the development of these new modified techniques and a mounting body of evidence in support of their efficacy, distal shunt procedures may eventually become the standard of care for treatment of refractory priapism.

Current literature describes arterial priapism in relation to patients who have had perineal or crural trauma that lacerates either the cavernosal artery or one of its branches causing an arteriolacunar fistula. Few idiopathic cases have been described [12]. There are a few reports of iatrogenic causes after treatment for a low-flow state [13–16] and there is one due to an iatrogenic laceration of the cavernosal artery [17]. This series describes two cases of high-flow priapism after surgical intervention to treat a low-flow state.

## 2. Case Presentation

### 2.1. Case 1

The first case is a 24-year-old Caucasian male that sought medical attention for priapism 24 hours after his erection began. He has a past medical history of stuttering priapism and idiopathic thrombocytopenic purpura diagnosed three years earlier but in remission according to his hematologist. His only previous surgery was a splenectomy. He was taking no medications at the time.

In the emergency room he received an intracorporal aspiration and injection of phenylephrine (500 *µ*g/mL) with no detumescence. A corporal blood gas was collected which showed the following: pCO2, 89 mmHg (normal = 32–43); pO2, 32 mmHg (normal = 83–108); pH 7.20 (normal = 7.37–7.44). He was taken to the operating room where bilateral Al-Ghorab shunts were performed. In the recovery unit, he had recurrence of his erection. The patient was then transferred to our facility for further management.

His physical exam on arrival revealed a persistent erection and with pain and he was taken to the operating room. Dark venous blood was drained from his corpora. A corpora cavernosal shunt (T-shunt) with tunneling was performed using #8 Hegar dilators. Detumescence was obtained intraoperatively. The procedure was completed and the patient was transferred to the PACU without complication, yet immediately postoperatively he again became erect. A corporal blood gas was taken, with the values consistent with a* high-flow* state: pCO2, 47 mmHg (normal = 32–43); pO2, 106 mmHg (normal = 83–108); pH 7.27 (normal = 7.37–7.44); and 98% 02 saturation. He was observed and discharged on post-op day 2, but he continued to have an erection for approximately three weeks without pain. He was seen at 6 weeks post-op and he was having normal erectile function.

### 2.2. Case 2

The second patient is a 33-year-old African American male with sickle cell disease who presented to a referring emergency room in a methamphetamine-induced psychotic state. While on a psychiatric hold in the ER, he developed an erection with some associated discomfort. Besides sickle cell disease, the patient's medical history includes anxiety, depression, opioid dependence, marijuana, cocaine, amphetamine, benzodiazepine, and opioid abuse, splenic autoinfarction. He has a 16-pack year smoking history and used illicit drugs twice weekly. His surgical history includes 2 Winter shunts in 2010 and 1 T-shunt in 2012. The patient has suffered from episodes of stuttering and ischemic priapism one to two times monthly for the past two years but noted a higher frequency of episodes over the past three months. These have usually resolved with intracorporal injections of phenylephrine that were somewhat painful. He finally agreed to be treated almost 24 hours later and received intracorporal injections of phenylephrine (0.5 mg/mL) bilaterally every few minutes. After the injection, there was no improvement of his condition. A corporal blood gas was collected and the values were as follows: pCO2, 84 mmHg (normal = 32–43); pO2, 39 mmHg (normal = 83–108); pH 7.21 (normal = 7.37–7.44).

He was taken to the operating room where a T-shunt was performed. There was minimal drainage on incision. A #8 Hegar dilator was used to dilate the corpora, irrigating a small volume of arterial blood with some mild improvement in his erection. Within 10 minutes of finishing the procedure the erection returned. A corporal blood gas was collected at that time and the values were as follows: pCO2, 45 mmHg (normal = 32–43); pO2, 105 mmHg (normal = 83–108); pH 7.35 (normal = 7.37–7.44). The patient was observed in the hospital for a week while being treated for his other medical issues. His erection resolved after 10 days and he was still having erectile function at 6 weeks post-op. The patient was seen in another hospital 2 months later and had a T-shunt and tunneling for priapism and had a similar erection in the recovery unit and was taken back to the operating room without a repeat blood gas for a proximal shunt. He later developed a urethral injury from his proximal shunt.

### 2.3. Outcomes

Both patients were seen in clinic 6–8 weeks following their procedures. Erectile function was assessed using IIEF-15 and physical examination. In both cases the high-flow erection resolved.

## 3. Discussion

Appropriate evaluation and management of priapism first requires the clinician to distinguish between ischemic and nonischemic states. This can be done based on the elements of the patient's history such as duration, degree of pain, previous priapism, drugs, trauma, and hematological disorders. Diagnostically, color duplex ultrasonography, and corporal blood gas values can help clarify the circulatory state of a patient's priapism.

Low-flow priapism is characterized by decreased blood flow through the corpora cavernosa leading to hypoxia, acidemia, and hypercapnia identified via corporal blood gas. A color duplex ultrasound of these patients shows a lack of circulation. Common causes of ischemic priapism can arise from hematological malignancies, sickle cell disease, or medications such as Phosphodiesterase Type 5 Inhibitors. In these patients, the fully tumescent corpora cavernosa are usually tender and painful.

High-flow priapism is usually caused by an arteriolacunar fistula leading to unregulated cavernous arterial blood flow but relatively normal venous outflow. These can be the result of crural or perineal trauma causing a laceration. These can also be the result of surgical interventions that create traumatic fistulas that lead to loss of regulation in blood flow. High-flow priapism patients generally present in a partially to fully erect state that may or may not be painful. Chronic, tolerated tumescence without full rigidity is highly suggestive of nonischemic priapism.

Patients with hematological abnormalities such as sickle cell disease are at risk for both high-flow and low-flow priapism [18], though they are most commonly seen with low-flow states due to occlusion of venous outflow. Sickle cell patients much like the patient in case 2 can suffer from stuttering, intermittent episodes of venoocclusive priapism that can either resolve or progress to ischemia. The mechanism, while not completely clear, is thought to involve microvascular occlusion by sickled erythrocytes [19]. Regardless, these patients are seen more frequently and thus undergo more procedures, which increase the risk of iatrogenic fistula formation. Furthermore, patients presenting with recurrent ischemic priapism can lead to a lapse in diagnostic rigor on the part of clinicians who might presume that the episode is yet another low-flow priapism. Such cases could see escalating interventions when first-line treatments like intracorporal injections and irrigations fail to control the erection.

In both of these cases, patients presented with low-flow priapism as demonstrated by their pain, duration, tumescence, and corporal blood gas. We hypothesize that the process of creating a distal shunt and tunneling inadvertently created an intracorporal fistula that converted their ischemic priapism to a high-flow state.

High-flow states have been reported in patients who have sickle cell disease [20] and have a history of recurrent priapism managed by intracorporal injections [21] and even after distal shunting procedures [22]. While patients with ischemic priapism not responding to first-line treatment should receive shunting, it is important to recognize that patients with recurrent or resistant priapism are exposed to a higher level of iatrogenic trauma to the corporal bodies. The patient in case 1 received a series of injections, an Al-Ghorab, and T-shunt in less than 36 hours, and, in case 2 our patient underwent frequent intracorporal irrigation and injection of phenylephrine to relieve his regularly occurring episodes of priapism. While limitations exist in our ability to draw definitive conclusions with a small sample size, the strength of our case report lies in identifying a complication of an increasingly popular procedure that is easily misidentified and may lead to further intervention. With the exact mechanism not known, it is possible that these patients could have been converted to a high-flow state during the tunneling portion of the distal shunting.

It is critical to identify the state of a patient's priapism throughout the treatment process to distinguish between low-flow states, where shunting is indicated, and high-flow states, where shunting is not indicated. Beyond the normal risks associated with surgery, the most common complication in shunting procedures is erectile dysfunction and thus should only be used if indicated.

However, high-flow priapism still remains a poorly understood complication for some patients with idiopathic or recurrent priapism. Both cases highlight the necessity for diagnostic testing before, during, and after the surgical management of a priapism episode alongside the patient's history and presentation to avoid unnecessary complications and improve outcomes for patients with priapism. Increased use of T-shunt with tunneling may increase the conversion of low-flow to high-flow states.

## References

[1] Gregory A., Broderick M. (2012). Priapism. *Campbell-Walsh Urology*.

[2] Montague D. K., Jarrow J. M., Broderick G. A. (2003). *Guidelines on the Management of Priapism*.

[3] Broderick G. A., Kadioglu A., Bivalacqua T. J., Ghanem H., Nehra A., Shamloul R. (2010). Priapism: pathogenesis, epidemiology, and management. *Journal of Sexual Medicine*.

[4] Ochoa Urdangarain O., Hermida Perez J. A. (1998). Priapism. Our experience. *Archivos Españoles de Urología*.

[5] Kandel G. L., Bender L. I., Grove J. S. (1968). Pulmonary embolism: a complication of corpus-saphenous shunt for priapism. *Journal of Urology*.

[6] Burnett A. L., Pierorazio P. M. (2009). Corporal “snake” maneuver: corporoglanular shunt surgical modification for ischemic priapism. *Journal of Sexual Medicine*.

[7] Brant W. O., Garcia M. M., Bella A. J., Chi T., Lue T. F. (2009). T-shaped shunt and intracavernous tunneling for prolonged ischemic priapism. *The Journal of Urology*.

[8] Zacharakis E., Raheem A. A., Freeman A. (2014). The efficacy of the T-shunt procedure and intracavernous tunneling (snake maneuver) for refractory ischemic priapism. *Journal of Urology*.

[9] Tabibi A., Abdi H., Mahmoudnejad N. (2010). Erectile function and dysfunction following low flow priapism: a comparison of distal and proximal shunts. *Urology Journal*.

[10] Segal R. L., Readal N., Pierorazio P. M., Burnett A. L., Bivalacqua T. J. (2013). Corporal burnett 'snake' surgical maneuver for the treatment of ischemic priapism: long-term followup. *Journal of Urology*.

[11] Shiraishi K., Matsuyama H. (2013). Salvage management of prolonged ischemic priapism: al-ghorab shunt plus cavernous tunneling with blunt cavernosotomy. *Journal of Sexual Medicine*.

[12] Singal R., Bawa A. S., Singh R., Sahu P., Gupta A. (2012). Surgical management of resistant priapism. *Indian Journal of Surgery*.

[13] McMahon C. G. (2002). High flow priapism due to an arterial-lacunar fistula complicating initial veno-occlusive priapism. *International Journal of Impotence Research*.

[14] Rodríguez J., Cuadrado J. M., Frances A., Franco E. (2006). High-flow priapism as a complication of a veno-occlusive priapism: two case reports. *International Journal of Impotence Research*.

[15] Lutz A., Lacour S., Hellstrom W. (2012). Conversion of low-flow to high-flow priapism: a case report and review (CME). *The Journal of Sexual Medicine*.

[16] Gottsch H. P., Berger R. E., Yang C. C. (2012). Priapism: comorbid factors and treatment outcomes in a contemporary series. *Advances in Urology*.

[17] Bertolotto M., Ciampalini S., Martingano P., Mucelli F. P. (2009). High-flow priapism complicating ischemic priapism following iatrogenic laceration of the dorsal artery during a winter procedure. *Journal of Clinical Ultrasound*.

[18] Birnbaum B. F., Pinzone J. J. (2008). Sickle cell trait and priapism: a case report and review of the literature. *Cases Journal*.

[19] Francis R. B., Johnson C. S. (1991). Vascular occlusion in sickle cell disease: current concepts and unanswered questions. *Blood*.

[20] Ramos C. E., Park J. S., Ritchey M. L., Benson G. S. (1995). High flow priapism associated with sickle cell disease. *The Journal of Urology*.

[21] Wallis C. J. D., Hoag N., Pommerville P. J., Huk M. E. (2009). Recurrent idiopathic high-flow priapism treated with selective arterial embolization after repeated initial treatments for low-flow priapism. *Journal of the Canadian Urological Association*.

[22] Seftel A. D., Haas C. A., Brown S. L., Herbener T. E., Sands M., Lipuma J. (1998). High flow priapism complicating veno-occlusive priapism: pathophysiology of recurrent idiopathic priapism?. *Journal of Urology*.

